# The effects of topical and intravenous JM-1232(-) on cerebral pial microvessels of rabbits

**DOI:** 10.1186/s12871-015-0016-x

**Published:** 2015-03-20

**Authors:** Kodai Ikemoto, Tadahiko Ishiyama, Noriyuki Shintani, Nobumasa Asano, Daniel I Sessler, Takashi Matsukawa

**Affiliations:** 1 Department of Anesthesiology, Faculty of Medicine, University of Yamanashi, 1110 Shimokato, Chuo Yamanashi, 409-3898 Japan; 2 Surgical Center, University of Yamanashi Hospital, University of Yamanashi, 1110 Shimokato, Chuo Yamanashi, 409-3898 Japan; 3 Department of Outcomes Research, The Cleveland Clinic, Ohio, USA

**Keywords:** Anesthesia, JM-1232(-), Cranial window, Cerebral microcirculation, Sevoflurane

## Abstract

**Background:**

JM-1232(-) is a novel anesthetic agent which acts through gamma-aminobutyric acid receptors. Cerebral pial vascular effects of JM-1232(-) are unknown. We thus evaluated topical and intravenous effects of JM-1232(-) on cerebral pial microvessels in rabbits, and the extent to which carbon dioxide (CO_2_) reactivity is preserved.

**Methods:**

Closed cranial windows were used to visualize cerebral pial circulation in 29 Japanese white rabbits. In the first experiment, the cranial window was superfused with increasing concentrations of JM-1232(-): 10^-11^, 10^-9^, 10^-7^, 10^-5^ mol/L, n = 8 per concentration. In the second experiment, we examined the effects of an intravenous bolus of 1 mg/kg bolus of JM-1232(-), followed by the continuous infusion at 0.3 mg/kg/minute on cerebral pial vascular alteration (n = 9). In the third, we examined CO_2_ reactivity of cerebral pial vessels under JM-1232(-) (n = 6) or sevoflurane anesthesia (n = 6).

**Results:**

Topical application of JM-1232(-) did not change pial venular diameter, and constricted arterials only at the highest concentration. Intravenous administration of JM-1232(-) produced cerebral pial constriction which gradually diminished over time. Under intravenous administration of JM-1232(-) and inhaled sevoflurane, diameters of vessels increased in parallel with CO_2_ partial pressure. Slopes of linear regression and correlation coefficients in arterioles and venules were comparable for JM-1232(-) anesthesia and sevoflurane anesthesia.

**Conclusions:**

Topical application of JM-1232(-) had little effect on cerebral pial vessels. Intravenous administration produced vasoconstriction of cerebral pial arterioles and venules, however those changes were clinically unimportant. In addition, JM-1232(-) did not impair CO_2_ responsiveness. At least from the perspective of vascular reactivity, JM-1232(-) thus appears safe for neurosurgical patients.

## Background

Benzodiazepines, which reduce both cerebral blood flow (CBF) and cerebral metabolic rate (CMR) proportionally [1,2], are often used for sedation and anesthesia in neurosurgical patients. JM-1232(-), a novel anesthetic agent, is sedative and hypnotic via activation of the benzodiazepine site of a gamma-aminobutyric acid type A (GABA_A_) receptors, although JM-1232(-) is not a benzodiazepine [3]. The drug appears to possess a wide therapeutic index and is thus promising as an intravenous anesthetic [4].

The relationship between benzodiazepines and CBF is well established. However, the direct in vivo effect of JM-1232(-) on cerebral pial vessels has not been evaluated previously, nor is the effect of intravenous administration on pial vessel diameter established. We therefore evaluated the *in vivo* effects of topical and intravenous JM-1232(-) on cerebral pial microvessels in rabbits using the cranial window technique. We also evaluated the effect of JM-1232(-) on carbon dioxide (CO_2_) reactivity of cerebral pial vessels.

## Methods

The Ethics Committee on Animal Research, University of Yamanashi, Yamanashi, Japan, approved this study. Experiments were performed on 29 Japanese white rabbits weighing 2.8-3.5 kg. An intravenous catheter was inserted into an ear vein and bicarbonate Ringer’s solution infused at 10 mL/kg/h. The animals were anesthetized with intravenous administration of pentobarbital sodium (20 mg/kg) or inhalation of sevoflurane. Anesthesia was maintained with an infusion of pentobarbital sodium (5 mg/kg/h) or sevoflurane (4%). Muscle relaxants were not used.

The animals were tracheostomized and their lungs were mechanically ventilated with oxygen using volume controlled ventilator ACE3000 (Acoma medical industry, Tokyo, Japan). Initial tidal volume was 30-40 ml/kg and respiratory rate was 20-30 beats/min. End-tidal CO_2_ (ETCO_2_) was continuously monitored (Vamos, Dräger medical, Tokyo, Japan). A catheter inserted to femoral artery allowed continuous monitoring of mean arterial blood pressure (MAP) and blood sampling. Based on ETCO_2_ measurements, the tidal volume and respiratory rate were adjusted to maintain arterial carbon dioxide partial pressure (PaCO_2_) between 30 and 50 mmHg. The animals’ core body temperature, rectal temperature, was maintained at 39 ± 1°C with a heating blanket.

A closed cranial window was used to visualize the cerebral pial microcirculation. Each rabbit was placed in the sphinx posture. The scalp was retracted and a 0.8-cm-diameter hole was made at the parietal bone. After bipolar coagulation of dural vessels, the dura and arachnoid membranes were cut and a thin ring of plastic was positioned over the hole and secured with bone wax and dental acrylic. The space under the window was filled with artificial cerebrospinal fluid (aCSF), and three polyethylene catheters were inserted into the plastic ring.

One catheter was attached to a reservoir bottle containing aCSF. The aCSF was suffused at 0.1 mL/min. Two other catheters served as an inlet for study drug solutions and an outlet for aCSF. The level of the outlet was maintained approximately 5-6 cm above the window to maintain intracranial pressure. The volume of fluid below the window was between 0.5 and 0.7 mL. The composition of aCSF was Na^+^ 151 mEq/L, K^+^ 3.5 mEq/L, Ca^2+^ 2.5 mEq/L, Mg^2+^ 1.3 mEq/L, HCO_3_^-^ 25 mEq/L, urea 40 mg/dL, and glucose 65 mg/dL [5].

The study was divided into three parts: the first evaluated the direct effects of JM-1232(-) on cerebral pial vessels, the second determined the effects of intravenous administration of JM-1232(-) on cerebral pial vessels, and the third considered the effect of JM-1232(-) on CO_2_ reactivity of cerebral pial vessels).

In the first experiment (n = 8), anesthesia was induced with intravenous administration of pentobarbital sodium (20 mg/kg), and was maintained with an infusion of pentobarbital sodium (5 mg/kg/h). JM-1232(-) (Maruishi Pharmaceutical, Osaka, Japan) was dissolved in aCSF to obtain concentrations of 10^-11^, 10^-9^, 10^-7^, 10^-5^ mol/L solutions. After a stabilization period, control values for cerebral pial arteriolar and venular diameters and various laboratory data including MAP, heart rate (HR), rectal temperature, arterial blood gas tensions and pH, plasma electrolytes, glucose and lactate concentration were recorded.

The cranial window was superfused with escalating concentrations of JM-1232(-) in aCSF. Superfusion rate was initially set at 30 mL/h for 2 min. Because space under the window was approximately 0.5 mL, superfusion rate at 0.5 mL/min for 2 min was sufficient to clear aCSF in the window. Subsequently, infusion rate was decreased to 6 mL/h for 5 min. This ensured a steady-state superfusion. We measured the diameters of cerebral pial vessels, MAP, HR, and rectal temperature, arterial blood gas tensions, pH, plasma electrolytes, glucose and lactate concentration once, 7 minutes after application of each JM-1232(-) concentration. The window was then flushed with aCSF for 30 minutes before administration of the next concentration, and the superfusion rate was set at 30 mL/h,

In the second set of experiment (n = 9), we examined the effects of intravenous administration of JM-1232(-) on cerebral pial vasculature. In a preliminary experiment, we determined that 1 mg/kg of JM-1232(-) followed by a continuous infusion at 0.3 mg/kg/min induced sleep and produced a bispectral index (BIS, processed EEG) of 70-80 which is consistent with moderate sedation. Data from the manufacturer indicates that the hypnotic dose of JM-1232(-) is 1 mg/kg for monkeys, corresponding to an effective plasma concentration of 600 ng/ml. The hypnotic dose for rats is 0.7 mg/kg, corresponding to an effective plasma concentration of 300 ng/ml. In rats, a continuous infusion of JM-1232(-) at 0.3 mg/kg/min produces plasma concentrations of at least 600 ng/mL within a few minutes. Plasma concentration-time curve shows accumulation effect and the steady-state concentration at 6 μg/ml is produced within 30 min. Moreover, plasma concentration of JM-1232(-) after stopping the continuous infusion at 0.3 mg/kg/min was maintained the hypnotic concentration 30 min or more. We thus used a bolus of 1 mg/kg JM-1232(-) followed by a continuous infusion at 0.3 mg/kg/min for the second and third set of studies.

Anesthesia was induced with sevoflurane, and was maintained with sevoflurane (4%). After a cranial window was established, sevoflurane was stopped. We confirmed that the end-tidal sevoflurane concentration decreased to almost 0% in 30 min. When animals started to move or 20 min after the discontinuation of sevoflurane, JM-1232(-) was given a bolus of 1 mg/kg intravenously, followed by a continuous infusion at 0.3 mg/kg/min. Control values for cerebral pial arteriolar and venular diameters and various laboratory data including MAP, HR, rectal temperature, arterial blood gas tensions and pH, plasma electrolytes, glucose and lactate concentration were recorded just before the start of JM-1232(-). The measurements of cerebral pial vascular diameters and laboratory examinations were performed 10, 20, 40, and 60 minutes after initiating the JM-1232(-) infusion. JM-1232(-) was then stopped, and the cerebral pial vascular diameters and laboratory data were obtained 5, 10, and 30 min after the discontinuation of JM-1232(-).

In the third set of experiment, we examined CO_2_ reactivity of cerebral pial vessels during intravenous administration of JM-1232(-) (n = 6) or inhaled sevoflurane (n = 6). In the JM-1232(-) anesthetized group, anesthesia was induced with JM-1232(-) 1 mg/kg bolus followed by 0.3 mg/kg/min. A cranial window was established. Then, control values for cerebral pial arteriolar and venular diameters and various laboratory data were recorded under normal ventilation (PaCO_2_ 40 ± 5 mmHg) at 0, 10, and 20 minutes. Hypercapnia (PaCO_2_: 50 ± 5 mmHg) was then induced by hypoventilation and cerebral pial vessel diameter and laboratory data were recorded at 0, 10, and 20 minutes. And finally, hypocapnia (PaCO_2_: 30 ± 5 mmHg) was induced by hyperventilation and measurements repeated at 0, 10, and 20 minutes. In the sevoflurane anesthetized group, anesthesia was induced and maintained with sevoflurane (4%). After cranial window implantation, pial vascular responses to hypocapnia (PaCO_2_, 30 ± 5 mmHg), normocapnia (PaCO_2_, 40 ± 5 mm Hg) and hypercapnia (PaCO_2_, 50 ± 5 mmHg) were studied as described above.

The diameters of several cerebral pial arterioles and venules were measured in each cranial window using a digital video analyzer (VH Analyzer VH-H1A5, Keyence, Osaka, Japan) on a personal computer that was attached to a microscope (VH-5000, Keyence, Osaka, Japan) via video capture unit (VH-E500, Keyence, Osaka, Japan). We selected diameters of arterioles and venules between 50 and 100 μm. Data from the pial views were stored on the computer hard disk for subsequent analysis after the experiments. MAP, HR, rectal temperature, arterial blood pH, PaCO_2_, arterial oxygen tension (PaO_2_), and plasma concentrations of Na^+^, K^+^, glucose and lactate in each experimental group, topical concentration-dependent effects of JM-1232(-), and CO_2_ responses of cerebral pial vessels were compared using analysis of variance (ANOVA) and *post hoc* Tukey tests. Cerebral pial vascular responses to topical JM-1232(-) were examined by paired *t*-test. Cerebral pial vascular responses to intravenous administration of JM-1232(-) were examined via ANOVA and Dunnett’s tests for *post hoc* comparisons. Responsiveness of CO_2_ data was assessed using linear regression, with slopes compared multivariably. Values are represented as means ± SDs; A *P* value less than 0.05 was considered statistically significant.

## Results

**Figure 1:**
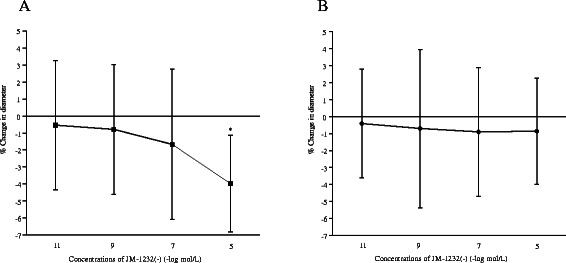
**The effects of topical application of JM-1232(-) on cerebral pial arteriolar (A) and venular (B) diameters.** Values are means ± SDs. Data are percent change in diameters compared with pre-drug measurements. JM-1232(-) at 10^-5^ mol/L constricted cerebral pial arterioles but had no effect on cerebral pial venules. (n = 8). * *P* < 0.001 compared with pre-application of JM-1232(-).

Body weight of the eight rabbits in the topical experiment was 3 ± 0.3 kg; it was 3.1 ± 0.1 kg in the nine rabbits of the intravenous administration of JM-1232(-) experiment, and 3 ± 0.1 kg in twelve animals for the CO_2_ response experiment. In the topical experiment, MAP, HR, rectal temperature, arterial pH, PaCO_2_, PaO_2_, base excess (BE) and plasma Na^+^, K^+^ glucose and lactate concentrations did not change significantly during the experimental period (Table 1). Absolute control diameter of arterioles was 76 ± 20 μm, and venules was 80 ± 23 μm. Topical application of JM-1232(-) at 10^-11^ to 10^-7^ mol/L did not produce any significant change on cerebral pial vessels. JM-1232(-) only at 10^-5^ mol/L induced constriction on cerebral pial arterioles (Figure 1A). In cerebral pial venules, JM-1232(-) did not exert any significant effect (Figure 1B).

**Table 1:** **Physiologic variables during topical application of JM-1232(-)**

JM-1232(-) concentration (mol /L) (n=8)								
	10^-11^pre	10^-11^post	10^-9^pre	10^-9^post	10^-7^pre	10^-7^post	10^-5^pre	10^-5^post
MAP (mmHg)	100 ± 5	100 ± 9	101 ± 10	102 ± 11	103 ± 11	101 ± 11	102 ± 11	102 ± 11
HR (bpm)	247 ± 12	251 ± 16	245 ± 17	246 ± 16	247 ± 18	245 ± 19	241 ± 19	245 ± 28
RT(**°**C)	39.5 ± 0.4	39.4 ± 0.4	39.4 ± 0.4	39.5 ± 0.5	39.5 ± 0.4	39.4 ± 0.5	39.4 ± 0.5	39.4 ± 0.5
pH	7.40 ± 0.05	7.40 ± 0.04	7.41 ± 0.03	7.40 ± 0.05	7.42 ± 0.02	7.42 ± 0.03	7.41 ± 0.03	7.41 ± 0.03
PaCO2 (mmHg)	43 ± 2	43 ± 3	43 ± 2	42 ± 2	41 ± 2	42 ± 2	42 ± 2	42 ± 3
PaO2 (mmHg)	472 ± 62	484 ± 43	489 ± 41	482 ± 54	482 ± 44	478 ± 66	498 ± 39	501 ± 35
Na^+^ (mEq/L)	142 ± 1	142 ± 1	142 ± 1	141 ± 1	142 ± 1	141 ± 1	141 ± 2	142 ± 2
K^+^ (mEq/L)	3.0 ± 0.4	3.0 ± 0.2	3.0 ± 0.2	3.0 ± 0.2	3.0 ± 0.2	3.0 ± 0.3	3.1 ± 0.3	3.0 ± 0.3
BS (mg/dl)	134 ± 18	133 ± 16	133 ± 10	132 ± 9	132 ± 10	130 ± 15	129 ± 9	128 ± 13
BE	2.4 ± 1.7	1.7 ± 2.3	1.4 ± 2.7	1.5 ± 2.6	1.8 ± 2.3	2.0 ± 2.1	1.6 ± 2.1	1.6 ± 2.1
Lactate (mmol/L)	1.5 ± 0.6	1.4 ± 0.5	1.5 ± 0.6	1.6 ± 0.7	1.6 ± 0.7	1.6 ± 0.7	1.7 ± 0.7	1.8 ± 0.6

Data are expressed as means ± SDs. There are no difference between each application period.

BS: blood glucose concentration, HR: heart rate, MAP: mean arterial pressure, RT: rectal temperature.

BE: Base excess.

**Figure 2:**
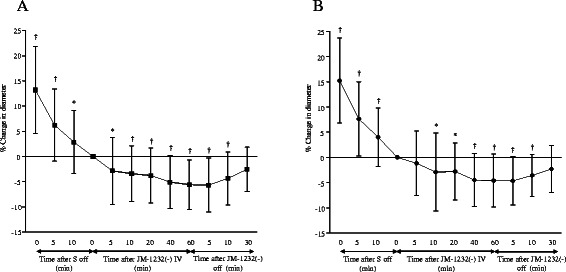
**The effects of intravenous administratin of JM-1232(-) on cerebral pial arteriolar (A) and venular (B) diameters.** (n = 9) Values are means ± SDs. Data are percent change in diameters compared with control (just before JM-1232(-) infusion). After discontinuation of sevoflurane, arteriolar and venular diameters constricted significantly. After intravenous administration of JM-1232(-), cerebral pial arteriolar constriction was induced at the time between 5 min and 60 min **(A)** and venular constriction was induced between 10 min and 60 min **(B)**. After discontinuation of JM-1232(-), cerebral pial arteriolar **(A)** and venular **(B)** constriction were attenuated. S = sevoflurane. * *P* < 0.05, † *P* < 0.01, compared with control.

In the experiment of intravenous administration of JM-1232(-), decreases in MAP and HR were observed, and lactate and glucose concentration increased during administration of JM-1232(-). However, rectal temperature, arterial pH, PaCO_2_, PaO_2_ and plasma Na^+^, K^+^ did not change significantly (Table 2). Absolute control diameter of arterioles was 61 ± 19 μm and venules was 66 ± 25 μm. Cerebral pial arterioles and venules were significantly constricted after discontinuation of sevoflurane (Figure 2A, B). Intravenous administration of JM-1232(-) induced cerebral pial arteriolar constriction at the time between 5 and 60 minutes (Figure 2A) and venular constriction between 10 and 60 minutes (Figure 2B). After discontinuation of JM-1232(-), constrictions of cerebral pial arterioles and venules were attenuated.

**Table 2:** **Physiologic variables during IV JM-1232(-) application (n = 9)**

	Pre	Soff10	control	JM10	JM40	JM60	JMoff10	JMoff30
MAP (mmHg)	82 ± 15	92 ± 13	94 ± 91	87 ± 12	83 ± 12*	78 ± 12*	82 ± 11*	90 ± 10
HR (bpm)	279 ± 15	273 ± 15	277 ± 21	260 ± 13	249 ± 27	239 ± 22*	272 ± 17	277 ± 16
RT(**°**C)	39.6 ± 0.8	39.6 ± 0.8	39.6 ± 0.7	39.6 ± 0.7	39.6 ± 0.7	39.6 ± 0.7	39.5 ± 0.6	39.6 ± 0.6
pH	7.44 ± 0.02	7.42 ± 0.03	7.41 ± 0.03	7.40 ± 0.03	7.40 ± 0.03	7.42 ± 0.03	7.39 ± 0.03	7.39 ± 0.03
PaCO2 (mmHg)	38 ± 3	40 ± 2	40 ± 2	41 ± 4	40 ± 3	40 ± 2	40 ± 2	40 ± 1
PaO2 (mmHg)	441 ± 38	455 ± 25	458 ± 27	456 ± 37	455 ± 33	451 ± 25	453 ± 28	454 ± 27
Na^+^ (mEq/L)	141 ± 1	141 ± 1	141 ± 1	142 ± 1	141 ± 1	142 ± 1	142 ± 2	142 ± 1
K^+^ (mEq/L)	3.8 ± 0.2	3.8 ± 0.2	3.8 ± 0.3	3.7 ± 0.2	3.7 ± 0.2	3.6 ± 0.1	3.7 ± 0.1	3.8 ± 0.2
BS(mg/dl)	123 ± 8*	122 ± 7*	128 ± 13	140 ± 12*	144 ± 13*	155 ± 19*	160 ± 19*	158 ± 20*
BE	2.3 ± 3.1	2.1 ± 2.7	2.2 ± 2.4	1.9 ± 2.6	1.9 ± 2.2	0.7 ± 2.6*	0.0 ± 2.8*	0.3 ± 2.1*
Lactate (mmol/L)	2.8 ± 0.7	2.9 ± 0.5	2.8 ± 0.4	3.4 ± 0.6*	4.2 ± 1.0*	5.0 ± 1.5*	5.4 ± 1.5*	5.2 ± 1.2*

Data are expressed as means ± SDs. *P < 0.05 compared with control.

S: sevoflurane, JM: JM-1232(-), Control: just before JM-1232(-) infusion, off 10: 10 min after sevoflurane discontinuation, JM 10, 40, and 60: 10, 40, 60 min after intravenous JM-1232(-) administration, ^JMoff 10, 30:^ 10 and 30 min after JM-1232(-) discontinuation, MAP: mean arterial pressure, HR: heart rate, RT: rectal temperature, BS: blood glucose concentration, BE: Base excess.

**Figure 3:**
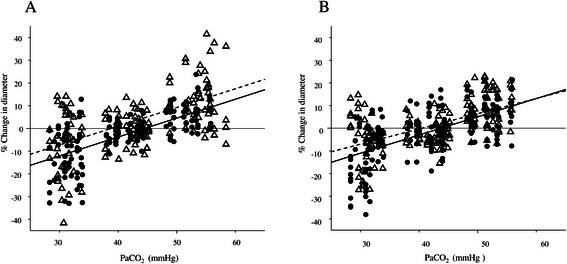
**Scatterplot and linear regression of cerebral pial arteriolar (A) and venular (B) CO**_**2**_**response in JM-1232(-) anesthetized (n = 6) and sevoflurane anesthetized rabbits.** (n = 6) Slopes of linear regression and correlation coefficients in arterioles and venules were comparable between JM-1232(-) anesthesia and sevoflurane anesthesia. Solid line shows the regression for JM-1232(-), and dashed line represents the regression for sevoflurane. ●JM-1232(-) anesthesia, ∆ sevoflurane anesthesia.

In the CO_2_ response experiment, HR, MAP, rectal temperature BE, PaO_2_, plasma Na^+^, K^+^, and lactate did not change significantly (Table 3). In the JM-1232(-) group, control diameter of arterioles was 62 ± 16 μm and venules was 74 ± 15 μm. In the sevoflurane group, control diameter of arterioles was 65 ± 17 μm and venules was 76 ± 11 μm. The percentage changes in the diameters of cerebral arterioles was similar with JM-1232(-) [% change in diameter = 0.83∙CO_2_ – 37, r = 0.67] and sevoflurane [% change in diameter = 0.82∙CO_2_ – 32, r = 0.56] as a function of CO_2_ partial pressure. Figure 3A shows that the slopes did not differ significantly. Likewise, the percentage change in diameters of cerebral venules was similar with JM-1232(-) [% change in diameter = 0.80∙CO_2_ – 35, r = 0.61] and sevoflurane [% change in diameter = 0.67∙CO_2_ – 27, r = 0.56] as a function of CO_2_ partial pressure. Figure 3B shows that the slopes did not differ significantly.

**Table 3:** **Physiologic variables during the study of CO**_**2**_ **reactivity on cerebral pial vessels under JM-1232(-) (n = 6) or sevoflurane anesthesia (n = 6)**

	JM Control	JM 40 ± 5	JM 50 ± 5	JM 30 ± 5	S Control	S 40 ± 5	S 50 ± 5	S 30 ± 5
MAP (mmHg)	72 ± 13	72 ± 8	69 ± 7	63 ± 6	99 ± 7	95 ± 4	98 ± 4	95 ± 7
HR (bpm)	249 ± 24	260 ± 14	272 ± 12	256 ± 4	242 ± 28	244 ± 45	248 ± 36	244 ± 43
RT(**°**C)	39.8 ± 0.3	39.7 ± 0.3	39.9 ± 0.2	40.1 ± 0.1	40.2 ± 0.5	40.2 ± 0.5	40.0 ± 0.7	40.1 ± 0.6
pH	7.42 ± 0.04	7.42 ± 0.05	7.32 ± 0.04†	7.54 ± 0.05†	7.40 ± 0.02	7.42 ± 0.03	7.33 ± 0.02*	7.51 ± 0.02*
PaCO2(mmHg)	42 ± 2	42 ± 2	55 ± 2†	31 ± 2†	41 ± 2	41 ± 2	50 ± 2*	32 ± 2*
PaO2 (mmHg)	451 ± 26	469 ± 15	437 ± 58	474 ± 25	480 ± 27	488 ± 32	476 ± 32	489 ± 34
Na^+^ (mEq/L)	140 ± 2	139 ± 3	140 ± 2	139 ± 2	141 ± 1	141 ± 1	142 ± 1	142 ± 2
K^+^ (mEq/L)	3.6 ± 0.4	3.7 ± 0.3	3.9 ± 0.4	3.9 ± 0.4	3.6 ± 0.4	3.6 ± 0.3	3.6 ± 0.2	3.4 ± 0.3
BS (mg/dl)	126 ± 15	119 ± 11†	120 ± 11	114 ± 8	135 ± 22	130 ± 15	135 ± 18	137 ± 12
BE	2.8 ± 4.0	3.0 ± 4.0	2.2 ± 2.7	2.8 ± 4.3	1.2 ± 2.0	1.2 ± 2.0	1.5 ± 2.2	1.0 ± 2.2*
Lactate (mmol/L)	1.7 ± 0.4	2.0 ± 0.6	2.3 ± 0.9	3.3 ± 1.0†	3.4 ± 2.0	3.2 ± 1.8	3.4 ± 1.9	4.1 ± 2.5

Data are expressed as means ± SDs. *P < 0.05 compared with S control. †*P* < 0.05 compared with JM control.

Control: the time PaCO_2_ 40 mmHg was confirmed by blood gas data, 30 ± 5, 40 ± 5, 50 ± 5: PaCO_2_ was 30 ± 5, 40 ± 5, and 50 ± 5 mmHg, JM:JM-1232(-), S: sevoflurane, MAP: mean arterial pressure, HR: heart rate, RT :rectal temperature, BS: blood glucose concentration, BE: Base excess.

## Discussion

Kuribayashi et al. reported that JM-1232(-) at between 10^-4^ and 5 x 10^-4^ mol/L depressed central respiratory activity, and estimated that the hypnotic dose was less than 10^-5^ mol/L [6]. As in rats, the effective concentration of JM-1232(-) in the rabbit is probably well under 10^-5^ mol/L. They also reported that the concentration of JM-1232(-) in the brain tissue after the injection of anesthetic dose may be lower than that of propofol in rats. We reported that clinically relevant propofol concentration in rabbits would be 1.2 x 10^-6^ mol/L or less [7]. Topical application of JM-1232(-) at concentrations of 10^-7^ to 10^-6^ mol/L, as in our study, thus correspond to clinically relevant therapeutic plasma concentrations in rabbits. This concentration of JM-1232(-), though, had no direct effect on cerebral pial vessels.

Topical JM-1232(-) had only slight constrictive effects on cerebral pial arterioles diameters, even at a concentration of at 10^-5^ mol/L. Our results contrast with those of Moriyama et al [8] who report that JM-1232(-) and midazolam produced dose-dependent relaxation in smooth muscle in human gastroepiploic artery in vitro. We previously reported that propofol had no effect on cerebral pial arteriolar or venular tone at clinical concentrations [7]. Nevertheless, propofol has direct effects upon systemic arterial and venous vascular tone [9], indicating that systemic and cerebral arterioles respond differently to some drugs.

At the higher concentrations of JM-1232(-), there may be significant penetration of the drug into the cerebral cortex and consequently systemic absorption. Furthermore, local absorption could locally inhibit neural transmission with consequent reductions in blood flow. Consistent with this theory, topical application of concentrated dexmedetomidine produced systemic actions and neutrally mediated cardiovascular effects [10].

Intravenous administration of JM-1232(-) constricted cerebral pial arterioles. Like benzodiazepines, JM-1232(-) has sedative and hypnotic properties that are mediated by benzodiazepine sites of GABA_A_ receptors [8]. Benzodiazepines such as midazolam and diazepam reduced both cerebral CBF and CMR [2]. CMR is associated with a proportional change in CBF. Our preliminary experiment and our previous study [11] showed that JM-1232(-) decreases BIS values. BIS is commonly used to measure hypnotic effect, and appears reliable in rabbits [12]. It therefore seems likely that JM-1232(-) decreased neural activity and CMR. As a result, CBF decreased and pial arterioles consequently constricted.

We also observed that intravenous administration of JM-1232(-) induced cerebral pial vasoconstriction that gradually reversed after discontinuation of the drug. Unpublished data from the pharmaceutical company indicate that plasma concentrations of JM-1232(-) are maintained at hypnotic concentrations was for at least 30 minutes after stopping a continuous infusion at 0.3 mg/kg/min. Presumably, hypnotic level decreased after stopping JM-1232(-), with consequent increases in CMR and CBF. Consistent with this theory, vasodilation of cerebral arterioles and an increase in CBF were observed when flumazenil was given to antagonize midazolam [13]. In addition, cerebral blood flow changed in parallel with cerebral pial arterial diameter changes [14]. JM-1232(-) thus appears to cause anesthesia which reduces metabolic rate, and therefore pial vessel flow when the drug is discontinued, anesthesia dissipates and pial flow returns.

In the third study, we examined CO2 responsiveness experiment under JM-1232(-) at 0.3 mg/kg/min intravenously and 1 MAC sevoflurane. Because CO_2_ responsiveness is well preserved under sevoflurane anesthesia [15], we used sevoflurane as a control. As expected from previous work, CO_2_ responsiveness was preserved under sevoflurane anesthesia in the present study. We demonstrated that arteriolar and venular linear regression slopes and correlation coefficients were comparable between JM-1232(-) anesthesia and sevoflurane anesthesia. Therefore, the responsiveness of the cerebral pial vessels to CO2 should be similar between JM-1232(-) anesthesia and sevoflurane anesthesia. Our results may be compatible with the previous result showing that CO2 responsiveness of the regional cerebral blood flow in the cerebral cortex preserved after the application of benzodiazepines such as midazolam [16]. We verified that CO2 responsiveness should be well preserved during anesthesia with JM-1232(-).

JM-1232(-) has sedative and anesthetic properties, however, sedative and anesthetic doses of JM-1232(-) for rabbits have not been well documented. Masamune et al. [11] reported that intravenous administration of JM-1232(-) at 0.01 mg/kg/min and 0.1 mg/kg/min combined with 0.2 minimum alveolar concentration of isoflurane reduced bispectral index (BIS) values in rabbits. However, BIS level at 0.01 mg/kg/min infusion and at 0.1 mg/kg/min infusion did not differ significantly [11]. Although the BIS algorithm of humans may not be applicable to rabbits, JM-1232(-) at 0.01 mg/kg/min probably has sedative property and that at 0.1 mg/kg/min has anesthetic property in rabbits if isoflurane would be inhaled. In our study, because we did not use isoflurane, we used BIS to determine hypnotic dose of JM-1232(-). As a result, we employed the higher dose of JM-1232(-) at 0.3 mg/kg/min. Shirasaka et al. [3] proposed that the intravenous administration of JM-1232(-) decreased MAP via activation of benzodiazepine GABA_A_ receptors. In addition, because MAP decreased after intravenous administration of JM-1232(-) at 0.3 mg/kg/min, benzodiazepine GABA_A_ receptors could have been activated effectively in our study. The sole application of JM-1232(-) at 0.3 mg/kg/min might produce sufficient anesthesia for rabbits.

We observed that intravenous administration of JM-1232(-) decreased MAP and decreased HR in rabbits. JM-1232(-) induced decrease in MAP attributed to a decrease in sympathetic nerve activity in rats [3] Moreover, Moriyama et al. reported that JM-1232(-) dose dependently relaxed smooth muscle in human gastroepiploic artery [8]. Decrease in MAP and HR caused by IV JM-1232(-) may be mediated by systemic vasodilation induced by blockade of sympathetic nerve activity and direct vasodilatory action. JM-1232(-) did not affect the baroreceptor reflex [3]. In baroreceptor reflex, the decrease in MAP results in an increase in HR. However, HR decreased after IV JM-1232(-). Blockade of sympathetic nerve activity caused by JM-1232(-) appears to overcome the baroreflex and as a result, HR decreased.

Plasma lactate concentrations were elevated in our second serious of experiments, but not in the third, although the same dose of JM-1232(-) was used in each. In the third series, anesthesia was induced and maintained with JM-1232(-) throughout the experiment. On the other hand, JM-1232(-) was started when animals started to move or 20 min after the discontinuation of sevoflurane in the second experiment. Animals may have suffered some stress at the period between discontinuation of sevoflurane and start of JM-1232(-) in the second experiment.

In the experiment of intravenous administration of JM-1232(-), when the end-tidal sevoflurane concentration decreased after discontinuation of sevoflurane, pial arterioles and venules were significantly constricted. This result indicates that sevoflurane *per se* has cerebrovascular dilator action on cerebral pial vessels. Our result concurs with Iida and coworkers’ who showed that sevoflurane significantly dilated cerebral pial arterioles in a concentration-dependent manner [17].

Cerebral circulation is regulated by metabolic regulatory mechanisms, chemical regulatory mechanisms, autoregulation, the autonomic nervous, and vascular endothelial regulation. Arterial pH, PaCO_2_, and PaO_2_ have substantial influences on cerebral vascular resistance and blood flow. Moreover, physiologic and pharmacologic stimuli may well have different upstream and downstream effects on the cerebral vasculature. Topical application of drugs into a cranial window allowed us to evaluate the direct effect of drugs without the changes in CMR and CBF consequent to systemic administration. Although venules actively dilate in response to topical nitroglycerin [18], topical JM-1232(-) did not alter venular diameter, suggesting that venules were passive conduits under the condition of our study.

We started JM-1232(-) when animals started to move or 20 min after the discontinuation of sevoflurane to avoid a period with no anesthesia. We thus do not have a true control in which vessel diameters were measured after sevoflurane was discontinued but without the addition of JM-1232(-). Beginning the JM-1232 infusion at 20 minutes thus may not reflect the true baseline for some animals.

## Conclusion

In conclusion, we evaluated the in vivo effects of JM-1232(-) on cerebral pial vessels in rabbits using the cranial window technique. Topical application of JM-1232(-) had little effect on cerebral pial vessels though high dose JM-1232(-) produced slight constriction. Intravenous administration produced vasoconstriction on cerebral pial arterioles and venules, however those changes were clinically trivial. And finally, CO_2_ responsiveness with JM-1232(-) was similar to that during sevoflurane anesthesia. At least from the perspective of vascular reactivity, JM-1232(-) thus appears safe for neurosurgical patients.

## Abbreviations

CO_2_: Carbon dioxide
CBF: Cerebral blood flow
CMR: Cerebral metabolic rate
GABA_A_: Gamma- aminobutyric acid type A
ETCO_2_: End-tidal CO_2_
MAP: Mean arterial blood pressure
PaCO_2_: Arterial carbon dioxide tension
aCSF: Artificial cerebrospinal fluid
HR: Heart rate
BIS: Bispectral index
PaO_2_: Arterial oxygen tension
ANOVA: Analysis of variance
BE: Base excess
